# Ixazomib Improves Bone Remodeling and Counteracts Sonic Hedgehog Signaling Inhibition Mediated by Myeloma Cells

**DOI:** 10.3390/cancers12020323

**Published:** 2020-01-30

**Authors:** Daniele Tibullo, Anna Longo, Nunzio Vicario, Alessandra Romano, Alessandro Barbato, Michelino Di Rosa, Ignazio Barbagallo, Carmelina Daniela Anfuso, Gabriella Lupo, Rosario Gulino, Rosalba Parenti, Giovanni Li Volti, Giuseppe Alberto Palumbo, Francesco Di Raimondo, Cesarina Giallongo

**Affiliations:** 1 Section of Biochemistry, Department of Biomedical and Biotechnological Sciences, University of Catania, 95123 Catania, Italy; d.tibullo@unict.it (D.T.); longo.anna@hotmail.it (A.L.); daniela.anfuso@unict.it (C.D.A.); gabriella.lupo@unict.it (G.L.); livolti@unict.it (G.L.V.); 2 Section of Physiology, Department of Biomedical and Biotechnological Sciences, University of Catania, 95123 Catania, Italy; nunziovicario@unict.it (N.V.); rosario.gulino@unict.it (R.G.); parenti@unict.it (R.P.); 3 Division of Hematology, Department of General Surgery and Medical-Surgical Specialties, A.O.U. “Policlinico-Vittorio Emanuele”, University of Catania, 95123 Catania, Italy; alessandrobarbato93@libero.it (A.B.); diraimon@unict.it (F.D.R.); cesarinagiallongo@yahoo.it (C.G.); 4 Section of Human Anatomy, Department of Biomedical and Biotechnological Sciences, University of Catania, 95123 Catania, Italy; mdirosa@unict.it; 5 Section of Biochemistry, Department of Drug Sciences, University of Catania, 95123 Catania, Italy; ignazio.barbagallo@unict.it; 6 Section of Haematology, Department of Medical and Surgical Sciences and Advanced Technologies “G.F. Ingrassia”, University of Catania, 95123 Catania, Italy

**Keywords:** multiple myeloma (MM), plasma cell (PC), ixazomib, bortezomib, mesenchymal stromal cell (MSC), sonic hedgehog (SHH), GLI1, osteoclastogenesis, osteoblastogenesis

## Abstract

Multiple myeloma (MM) is a clonal B-cell malignancy characterized by an accumulation of plasma cells (PC) in the bone marrow (BM), leading to bone loss and BM failure. Osteolytic bone disease is a common manifestation observed in MM patients and represents the most severe cause of morbidity, leading to progressive skeletal damage and disabilities. Pathogenetic mechanisms of MM bone disease are closely linked to PCs and osteoclast (OCs) hyperactivity, coupled with defective osteoblasts (OBs) function that is unable to counteract bone resorption. The aim of the present study was to investigate the effects of Ixazomib, a third-generation proteasome inhibitor, on osteoclastogenesis and osteogenic differentiation. We found that Ixazomib was able to reduce differentiation of human monocytes into OCs and to inhibit the expression of OC markers when added to the OC medium. Concurrently, Ixazomib was able to stimulate osteogenic differentiation of human mesenchymal stromal cells (MSCs), increasing osteogenic markers, either alone or in combination with the osteogenic medium. Given the key role of Sonic Hedgehog (SHH) signaling in bone homeostasis, we further investigated Ixazomib-induced SHH pathway activation. This set of experiments showed that Ixazomib, but not Bortezomib, was able to bind the Smoothened (SMO) receptor leading to nuclear translocation of GLI1 in human MSCs. Moreover, we demonstrated that PCs act as GLI1 suppressors on MSCs, thus reducing the potential of MSCs to differentiate in OBs. In conclusion, our data demonstrated that Ixazomib regulates bone remodeling by decreasing osteoclastogenesis and prompting osteoblast differentiation via the canonical SHH signaling pathway activation, thus, representing a promising therapeutic option to improve the complex pathological condition of MM patients.

## 1. Introduction

Multiple myeloma (MM) is a clonal B-cell malignancy characterized by accumulation of clonal plasma cells (PCs) in the bone marrow (BM), leading to bone loss and BM failure [1,2]. Pathogenetic mechanisms of MM bone loss are closely linked to MM PCs and osteoclasts (OCs) hyperactivity coupled with defective osteoblasts (OBs), which are unable to counteract bone resorption [3]. However, the molecular mechanisms underlying bone lesion in MM are still a matter of debate. Therefore, understanding the biological significance of this process would help in developing therapeutic strategies to control bone loss in MM patients. Recently, several inducers of bone lesion have been identified as targets for treating osteolytic complications, in addition to current treatments, including nitrogen-containing bisphosphonates and RANKL inhibitors [4]. Unfortunately, treatment with RANKL inhibitors can cause severe adverse effects, such as increased risk of infection, joint and muscle pain, uncontrolled serum calcium, osteonecrosis, and allergic reactions [5,6]. Furthermore, previous studies suggest that malignant PCs might themselves alter the cellular composition of the bone [7]. Indeed, PCs promote an osteoclastogenic effect by both exerting bone destruction and, throughout the recruitment, causing differentiation and activation of OC progenitors within the BM. In this scenario, the identification of proteasome as an essential machinery by which tumor cells regulate primary cellular processes, led to the introduction of proteasome inhibitors (PIs) as a breakthrough treatment for MM, in the clinical practice [8,9]. During osteoclastic differentiation, the binding of RANKL to RANK on the surface of osteoclast precursors activates NF-κB promoting osteoclast maturation and bone resorption [10]. Several authors demonstrated that bortezomib was able to inhibit NF-κB activation in osteoclasts, thus reducing osteoclastic differentiation and bone resorption [11,12,13]. To this regard, we have recently demonstrated that the proteasome inhibitor Bortezomib, commonly used to treat MM, inhibited osteoclastic differentiation by modulating the chitinase family genes [14]. We also observed that Bortezomib was able to inhibit osteoclast differentiation, thus, supporting the indication of Bortezomib in osteolytic MM [14]. Moreover, the increased bone resorption in MM patients is caused by impaired osteoblast differentiation. Several evidences suggest that MM cells interrupt several important signaling pathways such as the RANKL/OPG axis, which plays a key role in bone remodeling [15]. In such a context, PIs are able to significantly reduce RANKL expression levels, even if OPG were found to be not modulated by PIs treatment. Such a phenomenon induces a significant reduction of the RANKL/OPG ratio, thus, inhibiting MM bone disease [15].

Recently Ixazomib, an orally administrable third-generation PI, has been approved in the US and Europe, which has increased the therapeutic options for treating MM patients [16]. Previously reported evidences highlighted the molecular mechanisms underlying the activity of proteasome inhibitors as suppressors of osteoclastogenesis, thus, further supporting the therapeutic application of such agents in osteolytic MM [17,18]. Given the clinical importance of combined therapeutic approaches to reduce bone resorption in MM, we sought to investigate the role of PIs as concomitant suppressors of osteoclastogenesis and promoters of osteoblastogenesis. In this context, a crucial role in osteoblastogenesis is played by sonic hedgehog (SHH) signaling pathway [19]. SHH is a highly conserved pathway involved in patterning and morphogenesis of many organs in vertebrates [19] regulating OB differentiation and morphological transition [20,21,22]. SHH signaling is based on a 12-transmembrane protein SHH receptor Patched1 (PTCH1) that represses the 7-transmembrane protein Smoothened (SMO); following binding to its ligand SHH, PTCH1 is internalized and the SMO repression is alleviated, thus, inducing a complex and potent activation of the GLI-Kruppel family members (GLI1, GLI2, and GLI3) acting as transcription activators/repressors [23,24,25]. In particular, such cellular cascade is known as canonical SHH signaling pathway. Vice versa, non-canonical SHH signaling pathway does not rely on GLI-induced transcription modulation [26]. 

This study aimed at investigating the role of Ixazomib in modulating osteogenesis-related genes throughout SHH signaling pathway activation in the precursors of OCs and OBs, thus, further expanding the knowledge of the therapeutic effects of currently used compounds, as a treatment for MM patients with osteolytic complications. 

## 2. Results

### 2.1. Ixazomib Inhibits Osteoclastogenesis in Human Monocytes (MCs) Cultures under OC Differentiation Protocol

**Figure 1:**
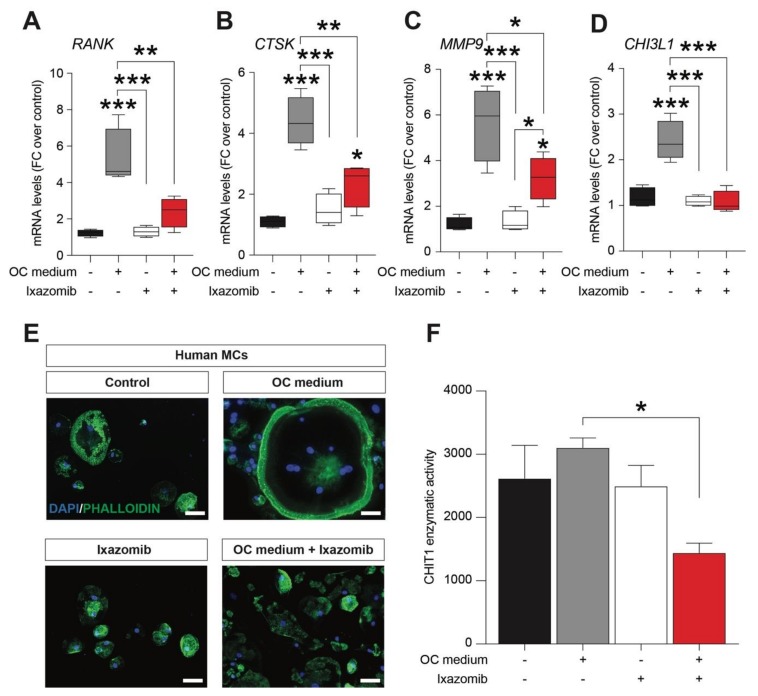
Ixazomib inhibits osteoclastogenesis in human monocyte (MCs) cultures under osteoclast (OC) differentiation protocol. qRT-PCR analysis of mRNA levels of the osteoclastogenic markers RANK (**A**), CTSK (**B**), MMP9 (**C**) and CHI3L1 (**D**) in MC cultures exposed to the OC medium, MCs treated with Ixazomib, and MCs exposed to the OC medium and treated with Ixazomib. Representative pictures of control MCs, MCs exposed to OC medium, MCs treated with Ixazomib and MCs exposed to the OC medium treated with Ixazomib (1 nM). DAPI (blue); PHALLOIDIN (green); scale bar: 50 μm (**E**). CHIT1 enzymatic activity in MC cultures exposed to the OC medium, MCs treated with Ixazomib, and MCs exposed to the OC medium and treated with Ixazomib (**F**). OC medium—osteoclastogenic medium. * *p*-value < 0.05, ** *p*-value < 0.01 and *** *p*-value < 0.001 vs. control or between groups.

In order to evaluate a potential effect of Ixazomib as inhibitor of osteoclastogenesis, we analyzed in vitro, the effects of Ixazomib in human monocyte (MCs) cultures, using a conventional osteoclastogenic differentiation protocol [10]. We first assessed the mRNA levels of osteoclastogenic differentiation markers, analyzing the TNF receptor superfamily member 11a (RANK), which is an essential mediator for osteoclast, and we found a strong upregulation in the OC medium exposed cultures (5.32 ± 0.1, mean ± SEM, *p*-value < 0.001 vs. control, Figure 1A). This increase was not observed in MCs treated with Ixazomib alone (1.31 ± 0.1, mean ± SEM, *p*-value = 0.14, Figure 1A), but Ixazomib was able to revert RANK increase in MCs exposed to the OC medium (2.38 ± 0.4, mean ± SEM, *p*-value = 0.286 vs. control, *p*-value = 0.003 vs. OC medium, Figure 1A). Similar effects were observed while analyzing the lysosomal cysteine proteinase involved in bone remodeling and resorption cathepsin K (CTSK), and in the matrix metallopeptidase 9 (MMP9) mRNA. Indeed, MCs showed a significant increase of CTSK (4.40 ± 0.4, mean ± SEM, *p*-value < 0.001 vs. control, Figure 1B) and MMP9 (5.66 ± 0.8, mean ± SEM, *p*-value < 0.001 vs. control, Figure 1C) in the OC medium. Conversely, Ixazomib treatment under differentiating conditions was able to significantly reduce CTSK levels (2.34 ± 0.4, mean ± SEM, *p*-value = 0.002 vs. OC medium, Figure 1B) and MMP9 levels (3.26 ± 0.5, mean ± SEM, *p*-value = 0.019 vs. OC medium, Figure 1C), as compared to the OC-medium-exposed MCs. We finally analyzed the level of the chitinase 3 like 1 (CHI3L1), which plays a crucial role in inflammation, tissue remodeling and in OC differentiation process. Consistently, we observed that the OC medium significantly increased the level of CHI3L1 (2.41 ± 0.2, mean ± SEM, *p*-value < 0.001 vs. control, Figure 1D), whereas Ixazomib alone was not able to induce upregulation of these markers in MCs, compared to the control cultures (2.34 ± 0.4, mean ± SEM, *p*-value = 0.9503 vs. control, Figure 1D). Interestingly, Ixazomib treatment in the OC medium condition inhibits the induction of the CHI3L1 gene expression (2.34 ± 0.4, mean ± SEM, *p*-value = 9503 vs. OC medium, *p*-value < 0.001 vs. OC medium Figure 1D). We further assessed the morphological pattern of MCs exposed to OC medium (Figure 1E), finding a characteristic morphological change at 21 days post differentiation induction (Figure 1E). Human MCs cells were differentiated into bona fide osteoclasts exhibiting significantly increased cell size and polynucleated cell bodies (Figure 1E). As expected, Ixazomib treated MCs under standard culture conditions did not show any detectable osteoclast-like structure (Figure 1E). Ixazomib in the cotreatment with OC differentiation medium was able to fully revert the osteoclastogenesis process, with undetectable polynucleated cells and osteoclast-like cells (Figure 1E). Finally, we analyzed the chitinase 1 (CHIT1) enzymatic activity, observing a significant reduction in the Ixazomib-treated OC-medium-exposed cultures (Figure 1F). 

### 2.2. Ixazomib Stimulates Osteoblastogenic Differentiation of Human Mesenchymal Stem Cells (MSCs)

**Figure 2:**
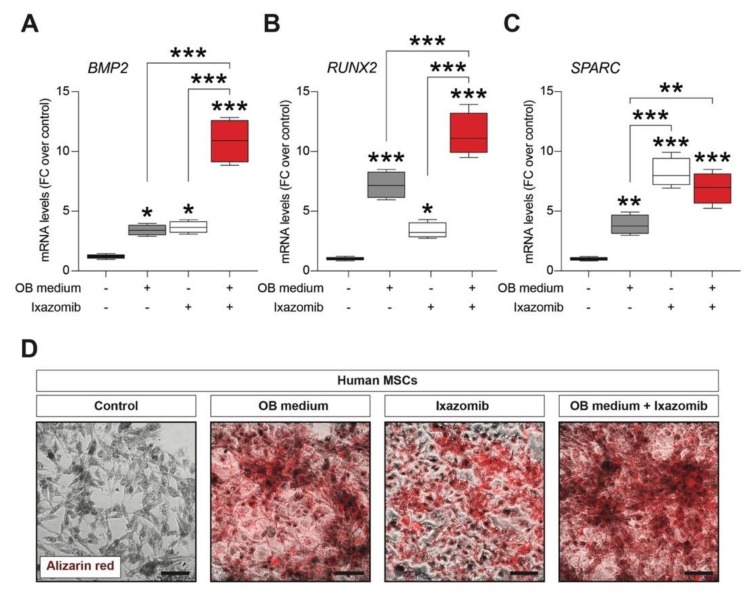
Ixazomib stimulates osteoblastogenic differentiation of human mesenchymal stem cells (MSCs). (**A**–**C**) qRT-PCR analysis of mRNA levels of the osteogenic markers BMP2 (**A**), RUNX2 (**B**), and SPARC (**C**) in MSC cultures exposed to the OB medium, MSCs treated with Ixazomib, and MSCs exposed to the OB medium and treated with Ixazomib. (**D**) Representative pictures of Alizarin red staining in control MSCs, MSCs exposed to the OB medium, MSCs treated with Ixazomib, and MSCs exposed to the OB medium treated with Ixazomib. Scale bar: 50 μm. OB medium—osteoblastogenic medium. * *p*-value < 0.05, ** *p*-value < 0.01 and *** *p*-value < 0.001 vs. control or between groups.

Given the role of Ixazomib as a robust suppressor of osteoclastogenesis on MCs, we sought to analyze the potential role of Ixazomib as a modulator of osteoblastogenesis on MSCs. To do so, we used the HS-5 (mesenchymal stroma cells, MSCs) cell line [27,28] and cultures were subjected to a 21 days differentiation protocol with the osteoblastogenic (OB) medium and the key markers of OBs were analyzed. Ixazomib treated control cultures and OB-medium-exposed cultures treated with Ixazomib were also included in the analysis. As expected, the MSC-cultures-exposed OB medium showed significantly increased mRNA levels of Bone morphogenetic protein 2 (BMP2) vs. the control MSCs (3.43 ± 0.2, mean ± SEM, *p*-value = 0.017 vs. control, Figure 2A). Notably, the Ixazomib treatment alone was able to significantly increase BMP2 mRNA levels in standard MSC culture conditions (3.66 ± 0.3, mean ± SEM, *p*-value = 0.013 vs. control, Figure 2A). Importantly, Ixazomib exerts a synergistic effect when delivered in cotreatment with the OB medium (*p*-value < 0.001 vs. OB medium and *p*-value < 0.001 vs. Ixazomib alone), increasing the levels of BMP2 mRNA of about 11 folds, as compared to the control cultures (10.87 ± 0.9, mean ± SEM, *p*-value < 0.001 vs. control, Figure 2A). We then analyzed the mRNA levels of two additional genes, the RUNX family transcription factor 2 (RUNX2), which is an essential OBs regulatory factor, and the secreted protein acidic and cysteine rich (SPARC), which encodes for a matrix-associated protein required for bone calcification. 

Both these markers were upregulated in the OB medium exposed MSC cultures (RUNX2: 7.19 ± 0.6, mean ± SEM, *p*-value < 0.001 vs. control; SPARC: 3.86 ± 0.4, mean ± SEM, *p*-value = 0.003 vs. control, Figure 2B,C). Notably, Ixazomib exposition for 21 days was sufficient to significantly increase both RUNX2 (3.37 ± 0.3, mean ± SEM, *p*-value = 0.012 vs. control, Figure 2B) and SPARC (8.21 ± 0.6, mean ± SEM, *p*-value < 0.001 vs. control, Figure 2C) mRNA levels. RUNX2 and SPARC were also found to be significantly upregulated in MSCs under the osteoblastogenic differentiation condition cotreated with Ixazomib (RUNX2: 11.41 ± 0.9, mean ± SEM, *p*-value < 0.001 vs. control; SPARC: 6.92 ± 0.7, mean ± SEM, *p*-value < 0.001 vs. control, Figure 2B,C).

We finally analyzed the potential of Ixazomib alone and in cotreatment with the OB medium to induce calcium deposits in MSC cultures. As shown in Figure 2D, a strong Alizarin red staining was detected in the OB-medium-exposed MSCs at 21 days of cultures. Of note, MSC cultures treated with Ixazomib or exposed to the osteoblastogenic differentiation protocol and cotreated with Ixazomib showed a clear accumulation of calcium deposits (Figure 2D).

### 2.3. Ixazomib, but Not Bortezomib, Binds Smoothened (SMO) Receptor in Human Mesenchymal Stem Cells (MSCs)

**Figure 3:**
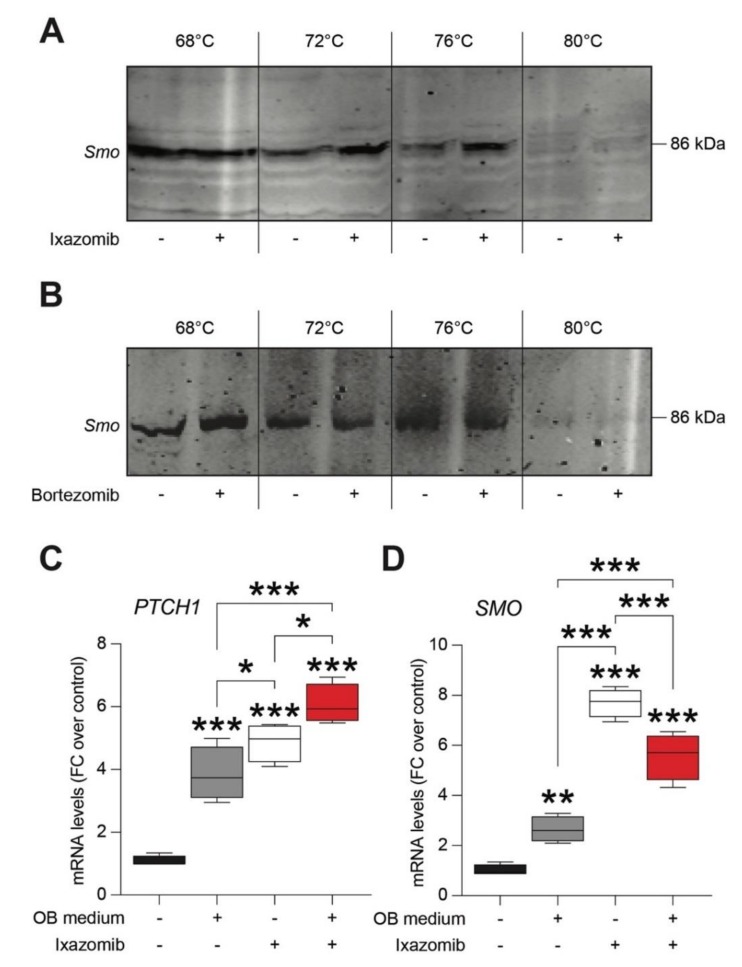
Ixazomib, but not Bortezomib, binds Smoothened (SMO) receptor in human mesenchymal stem cells (MSCs). (**A**) Representative blot of SMO analyzed by Cellular Thermal Shift Assay (CETSA) in MSCs protein content control and exposed to Ixazomib at 68 °C, 72 °C, 76 °C, and 80 °C. (**B**) Representative blot of SMO analyzed by CETSA in MSCs protein content control and exposed to Bortezomib at 68 °C, 72 °C, 76 °C, and 80 °C. (**C**,**D**) qRT-PCR analysis of mRNA levels of PTCH1 (**C**) and SMO (**D**) in MSC cultures exposed to the OB medium, MSCs treated with Ixazomib, and MSCs exposed to the OB medium and treated with Ixazomib. OB medium—osteoblastogenic medium. * *p*-value < 0.05, ** *p*-value < 0.01 and *** *p*-value < 0.001 vs. control or between groups.

Several evidences pinpointed a crucial role of SHH signaling in controlling cell fate, proliferation, and stem cells differentiation [29,30,31,32]. As such, we moved to study a potential mechanistic relation between Ixazomib-induced osteoblastogenesis stimulation and SHH signaling. Therefore, we compared the ability of Ixazomib and Bortezomib to bind and to stabilize SMO, the effector of the SHH intracellular signaling. Cellular thermal shift assay (CETSA) confirmed that Ixazomib, but not Bortezomib, was able to stabilize the SMO protein (Figure 3A,B), thus, suggesting that Ixazomib might act as an SMO ligand.

These results were further confirmed by qRT-PCR analysis on PTCH1 and G protein-coupled receptor SMO mRNA levels. Besides confirming a significant role of the SHH signaling during osteoblastogenesis (PTCH1: 3.85 ± 0.4, mean FC ± SEM, *p*-value < 0.001 vs. control; SMO: 2.65 ± 0.3, mean FC ± SEM, *p*-value = 0.003 vs. control; Figure 3C,D), we found that Ixazomib alone was able to significantly increase PTCH1 (4.87 ± 0.3, mean FC ± SEM, *p*-value < 0.001 vs. control, Figure 3C) and SMO (7.70 ± 0.3, *p*-value < 0.001 vs. control, Figure 3D) mRNA levels. Of note, the OB medium and Ixazomib, when in cotreatment, significantly increased the mRNA levels of both markers (PTCH1: 6.07 ± 0.3, mean FC ± SEM, *p*-value < 0.001 vs. control; SMO: 5.57 ± 0.5, mean FC ± SEM, *p*-value < 0.001 vs. control; Figure 3C,D).

### 2.4. Ixazomib Activates the Canonical GLI1-Dependent Sonic Hedgehog Signaling Pathway in MSCs inducing Osteoblastogenic Differentiation

**Figure 4:**
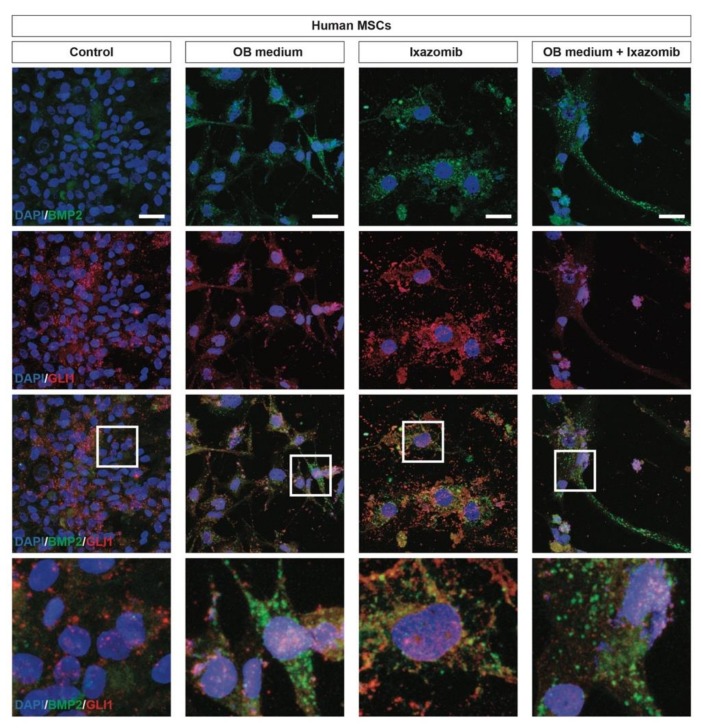
Ixazomib activates canonical GLI1-dependent Sonic Hedgehog (SHH) signaling pathway in MSCs inducing osteoblastogenic differentiation. Representative pictures of BMP2 (green) and GLI1 (red) immunostaining in control MSCs, MSCs exposed to the OB medium, MSCs treated with Ixazomib and MSCs exposed to the OB medium treated with Ixazomib. Scale bar: 25 μm. OB medium—osteoblastogenic medium.

We then investigated the differentiation profile of MSCs upon exposition to osteoblastogenic differentiation and Ixazomib treatment. We first assessed the expression of BMP2 in MSCs via immunofluorescence, finding a strong BMP2 upregulation and the characteristic morphological changes of differentiated MSCs, as compared to the untreated control cultures (Figure 4). We confirmed that the Ixazomib treatment was sufficient to induce a differentiation towards an OB phenotype in human MSCs (Figure 4). In order to assess the potential activation of the SHH pathway, we also analyzed the expression of the transcription factor GLI1, activated by canonical SMO-dependent SHH signaling. 

We found a robust nuclear translocation of GLI1 in differentiated human MSCs that were BMP2 positive (Figure 4), thus, indicating that the SHH pathway plays a key role in modulating stem cell fate and in inducing an osteoblastogenic differentiation. Of note, GLI1 was found to be extranuclear in undifferentiated human MSCs and treatment with Ixazomib was able to induce a SMO-dependent nuclear translocation of GLI1 (Figure 4).

### 2.5. MSCs Activates GLI1-Dependent SHH Signaling in OPM2 Cells

Given the evidences on the key role of canonical SHH signaling in modulating the fate of MSCs and differentiation, we established an in vitro coculture system using human MSCs and multiple myeloma cells. 

**Figure 5:**
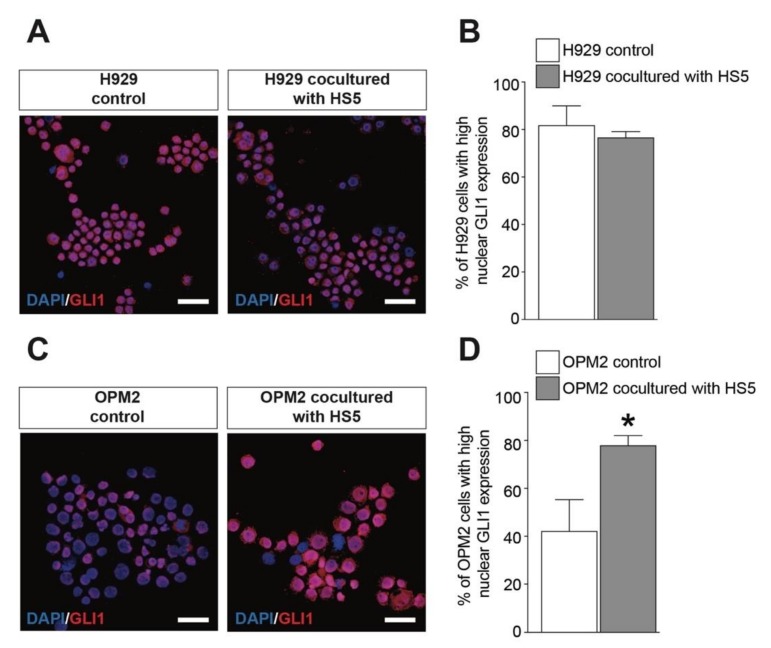
HS5 activates GLI1-dependent SHH signaling in OPM2 cells. (**A**,**B**) Representative pictures of H929 cells control and H929 cells cocultured with HS5 (**A**) and quantification of percentage of H929 cells expressing high nuclear GLI1 levels (**D**). (**C**,**D**) Representative pictures of OPM2 cells control and OPM2 cells cocultured with HS5 (**C**) and quantification of percentage of OPM2 cells expressing high nuclear GLI1 levels (**D**). Scale bar: 25 μm. * *p*-value < 0.05 vs. OPM2 control.

**Figure 6:**
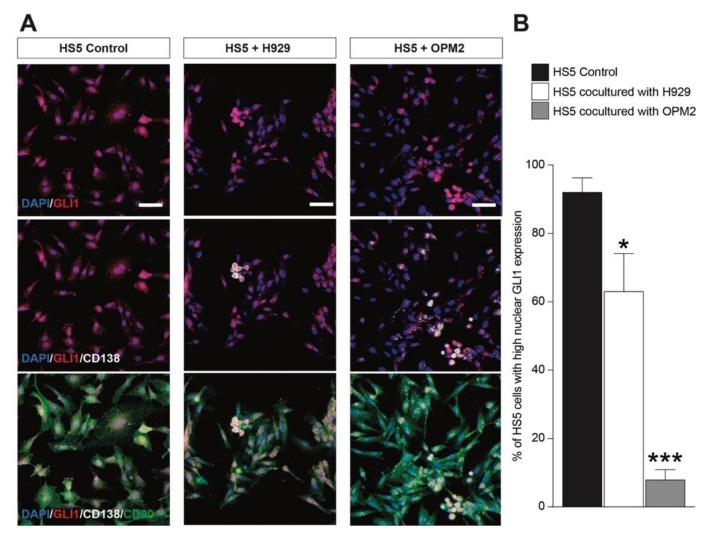
H929 and OPM2 cocultured with HS5 cells suppress nuclear GLI1 translocation. (**A**) Representative pictures of HS5 cells control, HS5 cells cocultured with H929 cells, and HS5 cells cocultured with OPM2 cells; cells were stained for GLI1 (red), CD138 (white, i.e., H929 or OPM2), and CD90 (green, i.e., HS5). (**B**) Quantification of percentage of HS5 cells (i.e., CD90 positive cells) expressing high nuclear GLI1 levels. Scale bar: 25 μm. * *p*-value < 0.05 and *** *p*-value < 0.001 vs. HS5 control.

We first assessed the nuclear translocation of GLI1 in H929 and OPM2 pre- and post-culture in the same well as that of the MSCs. We found that human H929 cells showed an increased nuclear localization of GLI1 and that the coculture with MSCs did not significantly alter the GLI1 translocation (Figure 5A,B). Importantly, OPM2 cells displayed a relatively low GLI1 activation (Figure 5C) that was observed upon same-well coculture with MSCs (Figure 5C,D). Concomitantly, we analyzed GLI1 activation on MSCs in control and post-cocultures with PCs. We found that both H929 and OPM2 cells (i.e., CD138 positive cells) were able to suppress the SHH signaling pathway on MSCs (i.e., CD90 positive cells, Figure 6A,B). These evidences support the hypothesis that myeloma cell lines reduce the SHH signaling pathway on MSCs, thus, inhibiting osteoblastogenic differentiation.

## 3. Discussion

Osteolytic bone disease is a central hallmark of MM, which severely impacts quality of life in patients [33,34]. PIs, such as Bortezomib, represent the cornerstones for combinatorial treatment of MM, and have shown significant effects in reducing bone resorption and promoting bone remodeling [9,35]. Such an effect has been proposed to be linked to the activity on nuclear factor kB (NF-kB) pathway and downregulation of CHIT1, CHI3L1, and MMP9, which are involved in both osteoclastogenesis and OC functioning [14]. Particularly, Bortezomib induces the stabilization of NF-kB antagonist I-kB and AP-1 transcription factors c-Fos and c-Jun, resulting in a reduction of osteoclast differentiation. Moreover, CHI3L1 has been shown to be targeted by Bortezomib and, besides being proposed as a prognostic marker for osteolytic complication in MM patients, it is a crucial modulator of mature OCs activity [14]. Given the increased therapeutic potential of third-generation PIs, we focused our attention on Ixazomib-induced effects on osteoclastogenesis and osteoblastogenesis. In this work we showed that Ixazomib was able to modulate the osteogenic and osteoclastic differentiation in vitro. Our data were in line with previously reported evidences on Bortezomib. We found that Ixazomib was able to inhibit the osteoclastogenic differentiation markers, such as RANK, MMP9, CTSK, and CHI3L1. Moreover, we observed a lower enzymatic activity of CHIT1 in MCs exposed to the OC medium and treated with Ixazomib. Such an effect might be linked to the Ixazomib inhibition on NF-kB and to a lower level of extracellular CHIT1 protein [14]. Indeed, Ixazomib strongly inhibits the differentiation of OCs from monocytes, thus, suggesting that therapeutic efficacy induced by Ixazomib benefits from the modulation of osteolytic cell populations. 

In order to evaluate the impact of Ixazomib on the potential osteogenic effects and the underlying molecular players involved in this scenario, we sought to modulate MSCs fate and differentiation upon osteogenic commitment. Interestingly, we found that Ixazomib treatment was able to activate key molecular regulators that pushed MSC differentiation, such as RUNX2, BMP2, and SPARC. Consistently, preclinical studies showed that clinically relevant concentrations of Ixazomib promote OB differentiation and inhibit OC formation in ex vivo experiments [36]. Furthermore, Yang et al., showed that Ixazomib was able to stimulate the parathyroid hormone (PTHR) inducing β-catenin signaling regulating the PTHR in OBs, which favors anabolic PTH action in bone [17,37]. Indeed, Wnt/β-catenin signaling pathway has been demonstrated to be activated by Ixazomib via β-catenin signaling via the cAMP/PKA-dependent pathway [37]. Notably, such evidences have also been previously reported for bortezomib and carfilzomib in Saos2 cells [38,39]. Our data suggest a potential mechanism in the modulation of bone remodeling mediated by Ixazomib treatment in vitro. Compelling evidences reported SHH signaling as crucial regulator of osteogenesis, at both, the embryonic stage and in adulthood, particularly to maintain bone mass [40,41,42]. Pharmacological and genetic modulation of PTCH1 has shown that disruption of SMO repression enhances osteoblastogenesis and osteoclastogenesis [40]. We tested the hypothesis that PIs might directly interact with the effector of SHH pathway SMO. Our evidences collectively demonstrate that Ixazomib, but not Bortezomib, was able to bind and activate SMO, leading to GLI1 nuclear translocation in MSCs and canonical SHH signaling pathway activation. This phenomenon was sufficient to induce a differentiation towards an OB phenotype, also in MSCs not exposed to differentiation media, and was synergistically effective when delivered in the MSCs under differentiation protocol. Interestingly, experimental evidences suggest that osteoblastogenesis activation was directly modulated by SMO activation and GLIs-induced signaling [41,43]. In particular, it has been proposed that different levels of modulation on the SHH signaling might significantly affect differentiation of precursor cells, as a full ablation of the signal showed dominance of bone resorption over bone formation, while partial or pharmacological modulation of the SHH pathway showed the opposite effects [41]. Moreover, our data and previously reported evidences suggest that PIs act as repressors of OC differentiation, also by their activity on other crucial pathways involved in cell fate determination [14]. Recent findings demonstrated that inhibition of SHH signaling using cyclopamine, an SMO antagonist, reduces osteoblastogenesis, and that knocking down PTCH1, which suppresses SMO activity, was beneficial for OCs differentiation.

The large body of evidences suggesting a crucial role of the SHH signaling in oncogenesis of B-cell malignancies and the potential of Ixazomib to induce SHH signaling, prompted us to assess the PCs–MSCs crosstalk leading to a reduction of osteoblastogenesis in MM patients. As for concerns about a possible link between these observations and SHH signaling, we demonstrated that PCs acts as GLI1 suppressors on MSCs, thus, reducing the potential of MSCs to differentiate in OBs. To this regard, previous reports highlighted a positive SHH autocrine loop on PCs, increasing their potential to spontaneously overcome stress conditions and apoptosis also protecting PCs from therapeutic approaches [44]. The SHH–GLI1–BLC-2 axis was found to inhibit PCs apoptosis, thus, reducing this activation might lead to sensitization against therapy [45]. In this context, the relative number proportion of PCs compared to endogenous MSCs plays a major role. Indeed, it is known that PCs outnumber MSCs in the MM microenvironment, therefore, our experimental conditions were set up to recapitulate this phenomenon resembling different cell interactions. MSCs, as other precursor cells, produced high amount of SHH that is intended for both autocrine stimulation and to communicate with bystander cell populations. In MM, a representative ratio between MSCs and PCs is about 1/100′000′1/1′000′000 [46,47]. This leads to a substantial deprivation of endogenous SHH stimulation on MSCs, thus, bending the differentiation of MSCs and reducing osteoblastogenesis [48,49]. As such, several groups have suggested stromal-derived SHH as a minor player in MM pathogenesis, even if further investigations are needed to better dissect this mechanisms [44,48]. Our data suggest that the axis between myeloma cell lines (i.e., H929 and OPM2) and MSCs leads to a suppression of the SHH signaling pathway in MSCs, thus, probably reducing the endogenous potential to compensate for osteolytic complications of MM. Ixazomib enhances in vitro MSCs differentiation into mature OBs and reduces osteoclastogenesis, paving the way for potential application of third-generation PIs in osteolytic complications of MM.

## 4. Materials and Methods 

### 4.1. Cell Lines and Cultures

Human monocytes (MCs) were isolated, after informed consent, from fresh buffy coat of healthy volunteers provided by the Transfusional Centre “Garibaldi” Hospital, Catania, S. ImmunoHaematology and Transfusional Medicine. MCs were then purified from the lymphomonocytic population by positive isolation, using magnetic beads coated with goat anti-mouse CD14^+^ IgG (cat. No. 130-050-201, Miltenyi Biotec GmbH, Bologna, Italy). MCs, identified as CD14^+^ and CD11c^+^ cells, showed a purity greater than 90% MCs isolated from PBMCs were cultured at a density of 5 × 10^5^ cells/cm^2^ in 24-well culture plates in MC mediums (2 mM gluta.mine, 10% FBS, 1% of P/S (Invitrogen, Milan, Italy) in conditioned Iscove’s Modified Dulbecco’s Media (IMDM) and cultured under standard culture conditions. 

Human H929 and OPM2 cell lines were obtained from American Type Culture Collection (Manassas, VA, USA). Cells were maintained in a PCs medium (2 mM glutamine, 10% FBS, 1% of P/S in RPMI medium), at a humidified 37 °C incubator providing 5% CO_2_. 

### 4.2. In Vitro Osteoclastogenic Differentiation

For in vitro osteoclastogenic differentiation, MCs were cultured in the OC medium [5 ng/mL rhRANK ligand (PeproTech. European Headquarters PeproTech, London, UK), 20 ng/mL rhM-CSF (PeproTech), 2 mM glutamine, 10% FBS, 1% of P/S in conditioned IMDM] for 21 days. A total of 10 nM of Ixazomib (MLN9708) was added to the cultures for 21 days. The medium was replaced every 3 days. 

### 4.3. In Vitro Osteoblastogenic Differentiation

Human HS-5 (MSCs) cell lines were cultured in the MSCs medium (10% fetal bovine serum (FBS), 1% of P/S (Invitrogen, Milan, Italy) in DMEM medium) [50,51]. Cells were plated at a final density of 5 × 10^5^ cells/cm^2^ and cultured either under standard culture conditions or exposed to osteoblastogenesis differentiation with OB medium for 21 days. A total of 10 nM of Ixazomib was added to the cultures at day 0, for the following 21 days.

### 4.4. Immunofluorescence

For immunofluorescence analysis, samples of paraformaldehyde (PFA)-fixed cells were incubated with blocking solution (10% normal goat serum (NGS), 0.1% Triton ×100 in PBS), for 1 h at room temperature [52]. Samples were then incubated overnight at 4 °C with the following primary antibody—rabbit anti-GLI1 (Afbcam Cambridge, UK), Cat# ab49314, RRID: AB_880198, 1:1000), mouse anti-BMP2 (Abcam Cambridge, UK, Cat# ab6285, RRID: AB_305401, 1:1000), mouse anti-CD138 (Abcam, Cat# ab34164, RRID: AB_778207, 1:1000), and rat anti-CD90 (Abcam, Cat# ab3105, RRID: AB_2287350, 1:1000).

The following day, samples were washed three times in 0.1% Triton X100 in PBS and then incubated for 1 h at room temperature with the appropriate combination of fluorescence conjugated secondary antibodies—goat polyclonal anti-mouse (Alexa Fluor 488, Thermo Fisher Scientific-Life Technologies, Monza Italy cat. No. A-11001, RRID: AB_2534069, 1:1000 or Alexa Fluor 546, Innovative Research (Thermo Fisher Scientific-Life Technologies, Monza, Italy) Cat. No. A21045, RRID: AB_1500928, 1:1000), goat polyclonal anti-rabbit (Alexa Fluor 488, Molecular Probes (Thermo Fisher Scientific-Life Technologies, Monza, Italy) Cat. No. A-11008, RRID: AB_143165, 1:1000 or Alexa Fluor 564, Molecular Probes Cat. No. A-11010, RRID: AB_143156, 1:1000), and goat polyclonal anti-rat (Alexa Fluor 647, Thermo Fisher Scientific Cat. No. A21247, RRID: AB_141778, 1:1000). Nuclei were counterstained with 4′,6-diamidino-2-phenylindole (DAPI, 1:1000, Cat# D1306, Invitrogen) for 5 min, at room temperature. Slices were mounted with fluorescent mounting medium Permafluor (Thermo Fisher Scientific) and digital images were acquired using a Leica DM IRB (Leica Microsystem, Buccinasco, Milan, Italy) fluorescence microscope or a Leica TCS SP8 confocal microscope. 

Phalloidin staining on the MCs was performed using Alexa Fluor 488 Phalloidin (Thermo Fisher Scientific Cat. No. A12379, RRID: AB_2759222, 1:200), incubated for 20 min at room temperature. Samples were then washed in 0.1% Triton X-100 in PBS and nuclei were counterstained with DAPI (1:1000) for 5 min, at room temperature. Slices were mounted and acquired as described above. For quantification of GLI1 and DAPI colocalization, nuclear ROIs were segmented and the corresponding GLI1 fluorescence intensity was quantified using ImageJ analysis software.

### 4.5. Alizarin Red Staining

Mineralization was determined using Alizarin Red S (Sigma, Milan, Italy) staining and phase-contrast microscopy, at 21 days of culture. Cells were incubated with 2% alizarin red with pH 4.2 for 10 min, subsequently washed with distilled water, and then observed with phase-contrast microscopy to examine cell morphology and to verify the presence of mineralized nodules.

### 4.6. CETSA

CETSA was used to investigate the target engagement of Ixazomib and Bortezomib (Velcade), as described previously [53,54,55]. Cells were suspended in PBS, at a final density of 5 × 10^6^ cells/mL, lysed via ultrasonication on ice, and treated with vehicle, 1 μM of Ixazomib or 1 μM Bortezomib for 1 h at 37 °C. The cell suspension was divided in 4 aliquots of 100 μL and heated for 3.5 min at 68, 72, 76, or 80 °C, respectively. Samples were centrifuged at 15,000× *g* for 20 min at 4 °C to separate the stable and denatured proteins, and supernatants were then collected and mixed with 4× Laemmli loading buffer and 10% β-mercaptoethanol, and incubated at 95 °C for 5 min. Proteins were separated on 4–20% Tris-glycine acrylamide gels (Thermo Scientific) and transferred to nitrocellulose membranes. Membranes were incubated for 1 h at room temperature with Odyssey blocking buffer solution, and then overnight at 4 °C with rabbit anti-SMO antibody (Abcam, Cat# ab72130, RRID: AB_1270802, 1:1000). After washes in 0.1% tween-20 in PBS, membranes were incubated for 1 h at room temperature with the secondary antibody (goat polyclonal anti-rabbit IRDye 680RD; LI-COR Biosciences, Cat# 926-68171, RRID: AB_10956389, 1:10,000). All antibodies were diluted in Odyssey blocking buffer solution. Proteins bands were imaged using an Odyssey Infrared Imaging Scanner (LI-COR Biosciences, Milan, Italy) and compared to the vehicle-treated controls.

### 4.7. qRT-PCR 

After RNA extraction and reverse transcription, samples were analyzed for expression of BMP2, RUNX2, SPARC, RANK, CTSK, MMP9, and CHI3L1 mRNA. Their expression was assessed by using 7900HT Fast Real-Time PCR System and TaqMan Universal PCR Master Mix (ThermoFisher, Monza, Italy). For each sample, the relative expression level of each studied mRNA was normalized using GAPDH as the invariant control. 

### 4.8. Statistical Analysis

All statistics were performed using GraphPad Prism (version 5.00 for Mac, GraphPad Software, San Diego, CA, USA). Data were tested for normality using a D’Agostino and Pearson omnibus normality test and subsequently assessed for homogeneity of variance. Data that passed both tests were further analyzed by two-tailed unpaired Student’s *t*-test for comparison of n = 2 groups. Comparisons of n > 2 groups were performed using a one-way ANOVA and Holm–Sidak’s multiple comparisons test. For all statistical tests, *p*-values < 0.05 were considered statistically significant; p-values are reported within the figure legends.

## 5. Conclusions

In conclusion, we found that Ixazomib was able to decrease osteoclastogenesis in MCs and concomitantly also increased MSCs osteogenic differentiation, throughout the activation of SMO/GLI1-dependent SHH signaling pathway. The relative importance of SHH signaling pathway in bone remodeling still need to be further investigated, to dissect the contribution of such a pathway in the pleiotropic mechanism of action of PIs in MM-derived cell lines. Moreover, our in vitro evidences uncover a novel axis between PCs and MSCs that leads to the suppression of the SHH signaling pathway in MSCs, thus, further reducing the endogenous potential to compensate for osteolytic complications of MM.

## References

[1] Kyle R.A., Rajkumar S.V. (2004). Multiple myeloma. N. Engl. J. Med..

[2] Palumbo A., Anderson K. (2011). Multiple myeloma. N. Engl. J. Med..

[3] Terpos E., Christoulas D., Gavriatopoulou M., Dimopoulos M.A. (2017). Mechanisms of bone destruction in multiple myeloma. Eur. J. Cancer Care.

[4] Tsubaki M., Komai M., Itoh T., Imano M., Sakamoto K., Shimaoka H., Takeda T., Ogawa N., Mashimo K., Fujiwara D. (2014). Nitrogen-containing bisphosphonates inhibit RANKL- and M-CSF-induced osteoclast formation through the inhibition of ERK1/2 and Akt activation. J. Biomed. Sci..

[5] Terpos E., Ntanasis-Stathopoulos I., Gavriatopoulou M., Dimopoulos M.A. (2018). Pathogenesis of bone disease in multiple myeloma: From bench to bedside. Blood Cancer J..

[6] Nishida H. (2018). Bone-targeted agents in multiple myeloma. Hematol. Rep..

[7] Noll J.E., Williams S.A., Tong C.M., Wang H., Quach J.M., Purton L.E., Pilkington K., To L.B., Evdokiou A., Gronthos S. (2014). Myeloma plasma cells alter the bone marrow microenvironment by stimulating the proliferation of mesenchymal stromal cells. Haematologica.

[8] Konstantinova I.M., Tsimokha A.S., Mittenberg A.G. (2008). Role of proteasomes in cellular regulation. Int. Rev. Cell Mol. Biol..

[9] Romano A., Conticello C., Di Raimondo F. (2013). Bortezomib for the treatment of previously untreated multiple myeloma. Immunotherapy-UK.

[10] Di Rosa M., Tibullo D., Vecchio M., Nunnari G., Saccone S., Di Raimondo F., Malaguarnera L. (2014). Determination of chitinases family during osteoclastogenesis. Bone.

[11] Zavrski I., Krebbel H., Wildemann B., Heider U., Kaiser M., Possinger K., Sezer O. (2005). Proteasome inhibitors abrogate osteoclast differentiation and osteoclast function. Biochem. Biophys. Res. Commun..

[12] Ang E., Pavlos N.J., Rea S.L., Qi M., Chai T., Walsh J.P., Ratajczak T., Zheng M.H., Xu J. (2009). Proteasome inhibitors impair RANKL-induced NF-kappaB activity in osteoclast-like cells via disruption of p62, TRAF6, CYLD, and IkappaBalpha signaling cascades. J. Cell Physiol..

[13] Von Metzler I., Krebbel H., Hecht M., Manz R.A., Fleissner C., Mieth M., Kaiser M., Jakob C., Sterz J., Kleeberg L. (2007). Bortezomib inhibits human osteoclastogenesis. Leukemia.

[14] Tibullo D., Di Rosa M., Giallongo C., La Cava P., Parrinello N.L., Romano A., Conticello C., Brundo M.V., Saccone S., Malaguarnera L. (2015). Bortezomib modulates CHIT1 and YKL40 in monocyte-derived osteoclast and in myeloma cells. Front. Pharmacol..

[15] Terpos E., Sezer O., Croucher P., Dimopoulos M.A. (2007). Myeloma bone disease and proteasome inhibition therapies. Blood.

[16] Zanwar S., Abeykoon J.P., Kapoor P. (2018). Ixazomib: A novel drug for multiple myeloma. Expert. Rev. Hematol..

[17] Yang Y., Blair H.C., Shapiro I.M., Wang B. (2015). The Proteasome Inhibitor Carfilzomib Suppresses Parathyroid Hormone-induced Osteoclastogenesis through a RANKL-mediated Signaling Pathway. J. Biol. Chem..

[18] Accardi F., Toscani D., Bolzoni M., Dalla Palma B., Aversa F., Giuliani N. (2015). Mechanism of Action of Bortezomib and the New Proteasome Inhibitors on Myeloma Cells and the Bone Microenvironment: Impact on Myeloma-Induced Alterations of Bone Remodeling. Biomed. Res. Int..

[19] Ingham P.W., McMahon A.P. (2001). Hedgehog signaling in animal development: Paradigms and principles. Genes Dev..

[20] Beachy P.A., Karhadkar S.S., Berman D.M. (2004). Tissue repair and stem cell renewal in carcinogenesis. Nature.

[21] Nakamura T., Naruse M., Chiba Y., Komori T., Sasaki K., Iwamoto M., Fukumoto S. (2015). Novel hedgehog agonists promote osteoblast differentiation in mesenchymal stem cells. J. Cell Physiol..

[22] Oliveira F.S., Bellesini L.S., Defino H.L., da Silva Herrero C.F., Beloti M.M., Rosa A.L. (2012). Hedgehog signaling and osteoblast gene expression are regulated by purmorphamine in human mesenchymal stem cells. J. Cell Biochem..

[23] Murone M., Rosenthal A., de Sauvage F.J. (1999). Hedgehog signal transduction: From flies to vertebrates. Exp. Cell Res..

[24] Briscoe J. (2006). Agonizing hedgehog. Nat. Chem. Biol..

[25] Alcedo J., Ayzenzon M., Von Ohlen T., Noll M., Hooper J.E. (1996). The Drosophila smoothened gene encodes a seven-pass membrane protein, a putative receptor for the hedgehog signal. Cell.

[26] Chen Y., Struhl G. (1996). Dual roles for patched in sequestering and transducing Hedgehog. Cell.

[27] Choi D.H., Suhaeri M., Hwang M.P., Kim I.H., Han D.K., Park K. (2014). Multi-lineage differentiation of human mesenchymal stromal cells on the biophysical microenvironment of cell-derived matrix. Cell Tissue Res..

[28] Liu C., Weng Y., Yuan T., Zhang H., Bai H., Li B., Yang D., Zhang R., He F., Yan S. (2013). CXCL12/CXCR4 signal axis plays an important role in mediating bone morphogenetic protein 9-induced osteogenic differentiation of mesenchymal stem cells. Int. J. Med. Sci..

[29] Fuchs S., Dohle E., Kirkpatrick C.J. (2012). Sonic Hedgehog-mediated synergistic effects guiding angiogenesis and osteogenesis. Vitam. Horm..

[30] Yang J., Andre P., Ye L., Yang Y.Z. (2015). The Hedgehog signalling pathway in bone formation. Int. J. Oral Sci..

[31] Iwasaki M., Kuroda J., Kawakami K., Wada H. (2018). Epidermal regulation of bone morphogenesis through the development and regeneration of osteoblasts in the zebrafish scale. Dev. Biol..

[32] Vicario N., Bernstock J.D., Spitale F.M., Giallongo C., Giunta M.A.S., Li Volti G., Gulisano M., Leanza G., Tibullo D., Parenti R. (2019). Clobetasol Modulates Adult Neural Stem Cell Growth via Canonical Hedgehog Pathway Activation. Int. J. Mol. Sci..

[33] Raje N., Roodman G.D. (2011). Advances in the biology and treatment of bone disease in multiple myeloma. Clin. Cancer Res..

[34] Anderson K., Ismaila N., Flynn P.J., Halabi S., Jagannath S., Ogaily M.S., Omel J., Raje N., Roodman G.D., Yee G.C. (2018). Role of Bone-Modifying Agents in Multiple Myeloma: American Society of Clinical Oncology Clinical Practice Guideline Update. J. Clin. Oncol..

[35] Romano A., Chiarenza A., Conticello C., Cavalli M., Vetro C., Di Raimondo C., Cunsolo R., Palumbo G.A., Di Raimondo F. (2014). Salvage therapy with pegylated liposomal doxorubicin, bortezomib, cyclophosphamide, and dexamethasone in relapsed/refractory myeloma patients. Eur. J. Haematol..

[36] Garcia-Gomez A., Quwaider D., Canavese M., Ocio E.M., Tian Z., Blanco J.F., Berger A.J., Ortiz-de-Solorzano C., Hernandez-Iglesias T., Martens A.C. (2014). Preclinical activity of the oral proteasome inhibitor MLN9708 in Myeloma bone disease. Clin. Cancer Res..

[37] Yang Y., Lei H., Qiang Y.W., Wang B. (2017). Ixazomib enhances parathyroid hormone-induced beta-catenin/T-cell factor signaling by dissociating beta-catenin from the parathyroid hormone receptor. Mol. Biol. Cell.

[38] Qiang Y.W., Hu B., Chen Y., Zhong Y., Shi B., Barlogie B., Shaughnessy J.D. (2009). Bortezomib induces osteoblast differentiation via Wnt-independent activation of beta-catenin/TCF signaling. Blood.

[39] Hu B., Chen Y., Usmani S.Z., Ye S., Qiang W., Papanikolaou X., Heuck C.J., Yaccoby S., Williams B.O., Van Rhee F. (2013). Characterization of the molecular mechanism of the bone-anabolic activity of carfilzomib in multiple myeloma. PLoS ONE.

[40] Mak K.K., Bi Y., Wan C., Chuang P.T., Clemens T., Young M., Yang Y. (2008). Hedgehog signaling in mature osteoblasts regulates bone formation and resorption by controlling PTHrP and RANKL expression. Dev. Cell.

[41] Lv W.T., Du D.H., Gao R.J., Yu C.W., Jia Y., Jia Z.F., Wang C.J. (2019). Regulation of Hedgehog signaling Offers A Novel Perspective for Bone Homeostasis Disorder Treatment. Int. J. Mol. Sci..

[42] Hojo H., Ohba S., Chung U.I. (2015). Signaling pathways regulating the specification and differentiation of the osteoblast lineage. Regen. Ther..

[43] Cannonier S.A., Sterling J.A. (2015). The Role of Hedgehog Signaling in Tumor Induced Bone Disease. Cancers.

[44] Liu Z., Xu J., He J., Zheng Y., Li H., Lu Y., Qian J., Lin P., Weber D.M., Yang J. (2014). A critical role of autocrine sonic hedgehog signaling in human CD138+ myeloma cell survival and drug resistance. Blood.

[45] Singh B.N., Fu J., Srivastava R.K., Shankar S. (2011). Hedgehog signaling antagonist GDC-0449 (Vismodegib) inhibits pancreatic cancer stem cell characteristics: Molecular mechanisms. PLoS ONE.

[46] Martin D.R., Cox N.R., Hathcock T.L., Niemeyer G.P., Baker H.J. (2002). Isolation and characterization of multipotential mesenchymal stem cells from feline bone marrow. Exp. Hematol..

[47] Haynesworth S.E., Goshima J., Goldberg V.M., Caplan A.I. (1992). Characterization of cells with osteogenic potential from human marrow. Bone.

[48] Cai K., Na W., Guo M., Xu R., Wang X., Qin Y., Wu Y., Jiang J., Huang H. (2019). Targeting the cross-talk between the hedgehog and NF-kappaB signaling pathways in multiple myeloma. Leuk. Lymphoma.

[49] Zavala G., Prieto C.P., Villanueva A.A., Palma V. (2017). Sonic hedgehog (SHH) signaling improves the angiogenic potential of Wharton’s jelly-derived mesenchymal stem cells (WJ-MSC). Stem Cell Res. Ther..

[50] Calabrese G., Giuffrida R., Forte S., Fabbi C., Figallo E., Salvatorelli L., Memeo L., Parenti R., Gulisano M., Gulino R. (2017). Human adipose-derived mesenchymal stem cells seeded into a collagen-hydroxyapatite scaffold promote bone augmentation after implantation in the mouse. Sci. Rep..

[51] Calabrese G., Giuffrida R., Fabbi C., Figallo E., Lo Furno D., Gulino R., Colarossi C., Fullone F., Giuffrida R., Parenti R. (2016). Collagen-Hydroxyapatite Scaffolds Induce Human Adipose Derived Stem Cells Osteogenic Differentiation In Vitro. PLoS ONE.

[52] Vicario N., Pasquinucci L., Spitale F.M., Chiechio S., Turnaturi R., Caraci F., Tibullo D., Avola R., Gulino R., Parenti R. (2019). Simultaneous Activation of Mu and Delta Opioid Receptors Reduces Allodynia and Astrocytic Connexin 43 in an Animal Model of Neuropathic Pain. Mol. Neurobiol..

[53] Gulino R., Vicario N., Giunta M.A.S., Spoto G., Calabrese G., Vecchio M., Gulisano M., Leanza G., Parenti R. (2019). Neuromuscular Plasticity in a Mouse Neurotoxic Model of Spinal Motoneuronal Loss. Int. J. Mol. Sci..

[54] Paratore S., Parenti R., Torrisi A., Copani A., Cicirata F., Cavallaro S. (2006). Genomic profiling of cortical neurons following exposure to beta-amyloid. Genomics.

[55] Almqvist H., Axelsson H., Jafari R., Dan C., Mateus A., Haraldsson M., Larsson A., Martinez Molina D., Artursson P., Lundback T. (2016). CETSA screening identifies known and novel thymidylate synthase inhibitors and slow intracellular activation of 5-fluorouracil. Nat. Commun..

